# A comparative study of time-specific oxidative stress after acute myocardial infarction in patients with and without diabetes mellitus

**DOI:** 10.1186/s12872-016-0259-6

**Published:** 2016-05-23

**Authors:** Daisuke Kitano, Tadateru Takayama, Koichi Nagashima, Masafumi Akabane, Kimie Okubo, Takafumi Hiro, Atsushi Hirayama

**Affiliations:** Division of Cardiology, Department of Medicine, Nihon University School of Medicine, 30-1 Oyaguchi-kamicho, Itabashi-ku, Tokyo, 173-8610 Japan

**Keywords:** Derivatives of reactive oxygen metabolite, Oxidative stress, Acute myocardial infarction, Diabetes mellitus

## Abstract

**Background:**

Oxidative stress is involved in the initiation and progression of atherosclerosis, and hyperglycemia is known to increase oxidative stress, which injures the endothelium and accelerates atherosclerosis. To clarify the relation between oxidative stress, diabetes mellitus (DM), and acute myocardial infarction (AMI), we evaluated and compared time-specific oxidative stress after AMI in patients with and without DM by simple measurement of derivatives of reactive oxygen metabolites (d-ROMs) levels as indices of reactive oxygen species production.

**Methods:**

Sixty-eight AMI patients were enrolled (34 non-DM patients and 34 DM patients). Using the FRAS4 free radical analytical system, we measured d-ROMs levels in each patient at two time points: 1 and 2 weeks after AMI onset.

**Results:**

d-ROM levels decreased significantly between week 1 and week 2 (from 475.4 ± 119.4 U.CARR to 367.7 ± 87.9 U.CARR, *p* < 0.001) in the non-DM patients but did not change in the DM patients (from 463.1 ± 109.3 U.CARR to 461.7 ± 126.8 U.CARR, *p* = 0.819). Moreover, significant correlation was found in the total patient group between d-ROMs levels at 1 week and N-terminal prohormone of brain natriuretic peptide (r = 0.376, *p* = 0.041) and between d-ROM levels at 2 weeks and 2-hour oral glucose tolerance test glucose levels (r = 0.434, *p* < 0.001).

**Conclusions:**

Exposure to oxidative stress is greater in AMI patients with DM than AMI patients without DM. Our study results suggest that it is the continuous hyperglycemia that increases oxidative stress in these patients, causing endothelial dysfunction and accelerating atherosclerosis. However, long-term follow up study is needed to assess whether the increased oxidative stress affects patient outcomes.

**Electronic supplementary material:**

The online version of this article (doi:10.1186/s12872-016-0259-6) contains supplementary material, which is available to authorized users.

## Background

Oxidative stress, which is implicated in various disorders, especially lifestyle-related diseases such as diabetes mellitus (DM), is involved in the initiation and progression of atherosclerosis [1–3]. Biomarkers of oxidative stress, such as serum malondialdehyde measured as thiobarbituric acid-reactive substances, oxidized low-density lipoprotein, oxidative DNA damage byproduct 8-hydroxydeoxyguanosine, and urinary 8-iso-prostaglandin-F2α, are generally measured in research laboratories [4–7]. Indices of antioxidant potential, especially intracellular levels of superoxide dismutase and glutathione peroxidase, are also measured [8, 9]. However, the assay methods are complex and not suitable for large-scale analysis. Simpler means of detecting reactive oxygen species (ROS) by assay of derivatives of reactive oxygen metabolites (d-ROMs) and biological antioxidant potential have been developed, and reports of these methods and studies based on these methods have been increasing [10–19].

DM is a major risk factor for cardiovascular disease. Continuous hyperglycemia increases advanced glycation end products (AGEs) and induces ROS production [20]. ROS plays a pivotal role in the development of the microvascular and cardiovascular complications of DM. The increased ROS in patients with type 2 DM and metabolic syndrome is a consequence of metabolic abnormalities, including hyperglycemia, insulin resistance, hyperinsulinemia, and dyslipidemia [21], each of which contributes to mitochondrial superoxide overproduction in endothelial cells of large and small vessels as well as the myocardium [22]. The atherosclerosis is accelerated and may induce acute coronary syndrome.

Thus, we are interested in the relations between oxidative stress, DM, and acute myocardial infarction (AMI), we evaluated and compared time-specific oxidative stress after AMI in patients with and without DM by simple measurement of d-ROMs levels as indices of ROS production.

## Methods

### Study patients

The study involved 68 consecutive patients who had suffered an ST-elevated AMI, admitted to the coronary care unit of Nihon University Itabashi Hospital between April 2010 and March 2011, and underwent successful primary percutaneous coronary intervention (PCI). Thirty-four had type 2 DM (DM group) and 34 did not (non-DM group). Patients with severe MI; those recovering from cardiopulmonary arrest or heart failure; and those with cardiomyopathy, severe valvular disease, atrial fibrillation, chronic kidney disease requiring hemodialysis, type 1 DM, type 2 DM requiring insulin or glucagon-like peptide-1 receptor agonists treatment, collagen disease, or malignant tumor were excluded from the study. Medications patients had been taking were not changed, and anti-diabetic agents were not given during the study period. We also collected d-ROM values in stable coronary artery disease (CAD) patients without DM (n = 40) and with DM (n = 28) as reference value, they had undergone coronary stenting for stable CAD except acute coronary syndrome more than 8 months before data collection.

The study was approved by the ethics committee of Nihon University Itabashi Hospital, and written informed consent was provided by each patient for participation.

### Clinical evaluation and laboratory measurements

Patients’ clinical characteristics, including age, sex, body mass index, smoking history, and history of hypertension and dyslipidemia were recorded, and blood samples were drawn 1 week after AMI onset for measurement of hemoglobin A1c (HbA1c), fasting glucose, total cholesterol, low-density lipoprotein-cholesterol, high-density lipoprotein-cholesterol, creatine phosphokinase (CPK), estimated glomerular filtration rate (eGFR), high-sensitivity C-reactive protein (hs-CRP) and N-terminal prohormone of brain natriuretic peptide (NT-proBNP). Insulin resistance was evaluated by means of homeostasis model assessment of insulin resistance (HOMA-IR), and an oral glucose tolerance test (OGTT) was given to evaluate glucose clearance.

### Assay of oxidative stress

We quantified hydrogen peroxide levels by measuring d-ROMs using the FRAS4 Free Radical Analytical System (H&D srl, Parma, Italy). Hydrogen peroxides are converted into radicals that oxidize N, N-diethyl-para-phenylenediamine and can be detected spectrophotometrically with the use of an all-purpose automatic analyzer. The d-ROM levels are expressed in arbitrary units called Carratelli units (U.CARR) [10]. The normal reference level of d-ROMs is 250 to 300 U.CARR [23, 24]. We measured d-ROMs at 2 time points, 1 week and 2 weeks after AMI onset, to avoid the possible influence of AMI.

### Statistical analysis

Continuous variables are expressed as mean ± SD values, and categorical variables are presented as numbers and percentages. Between-group differences were analyzed by one-way ANOVA with Tukey post-hoc honest significant difference test or by chi-square test, as appropriate. Differences between 1-week and 2-week values were analyzed by paired *t*-test, Wilcoxon signed-rank test, or unpaired *t*-test, as appropriate. Association between d-ROM levels and clinical variables was tested by linear regression analysis, and factors predictive of no or little change in the d-ROM level between 1 and 2 weeks after AMI were identified by multiple logistic regression analysis. Statistical analyses were performed with JMP ver. 9 (SAS Institute, Cary, NC, USA). A *p* value of 0.05 was considered significant.

## Results

### Patients’ clinical characteristics and laboratory values

Clinical characteristics of the study patients are shown in Table 1. Only HbA1c, fasting plasma glucose, 2-h OGTT glucose, HOMA-IR, eGFR, and the use of nitrate differed significantly between the two patient groups. Additional file 1 shows clinical characteristics of reference patients.

**Table 1:** Clinical characteristics of study patients upon enrollment^a^, per study group

	non-DM	DM	*p* value^b^
	*n* = 34	*n* = 34	
Age (years)	62.1 ± 9.6	61.4 ± 9.9	0.739
Sex, male (%)	32 (94.1)	29 (85.3)	0.259
BMI	24.7 ± 2.5	25.3 ± 2.6	0.293
Risk factors			
Hypertension, n (%)	28 (82.4)	29 (85.3)	0.742
Dyslipidemia, n (%)	26 (76.5)	26 (76.5)	1.000
Smoking, n (%)	19 (55.9)	23 (67.6)	0.318
Biochemical markers			
HbA1c (%)	5.6 ± 0.3	6.6 ± 0.5	**<0.001**
Fasting plasma glucose (mg/dL)	97.5 ± 7.4	114.6 ± 18.6	**<0.001**
2 h OGTT plasma glucose (mg/dL)	119.8 ± 13.0	172.7 ± 33.7	**<0.001**
HOMA-IR	1.53 ± 0.81	2.73 ± 1.89	**<0.001**
eGFR (mL/min/1.73 m^2^)	63.2 ± 16.0	71.7 ± 16.5	**0.035**
CPK (maximum) (U/L)	3532.2 ± 3898.3	2360.6 ± 2181.8	0.247
hs-CRP (mg/dL)	0.654 ± 0.695	0.828 ± 0.712	0.311
Total cholesterol (mg/dL)	199.9 ± 35.3	196.0 ± 57.4	0.954
Triglyceride (mg/dL)	95.0 ± 55.2	127.6 ± 77.8	0.277
HDL cholesterol (mg/dL)	46.6 ± 12.3	45.8 ± 10.0	0.825
LDL cholesterol (mg/dL)	134.3 ± 29.5	116.7 ± 37.0	0.752
NT-proBNP (pg/mL)	804.8 ± 1057.8	645.1 ± 915.0	0.578
Medications			
Ca channel blocker, n (%)	9 (26.5)	6 (17.6)	0.470
Beta blocker, n (%)	19 (55.9)	18 (52.9)	0.924
ACE-I/ARB, n (%)	22 (64.7)	25 (73.5)	0.504
Nitrate, n (%)	30 (88.2)	18 (52.9)	**0.004**
Statin, n (%)	24 (70.6)	23 (67.6)	0.853

^a^At 1 week after AMI onset

Data are expressed as mean ± SD or number (%). ^b^obtained by ANOVA or chi-square test. HbA1c, fasting plasma glucose, 2 h OGTT plasma glucose, HOMA-IR and eGFR levels were significantly higher and the use of nitrate was signifincatly lower in DM patients group.  *DM* diabetes mellitus, *BMI* body mass index, *HbA1c* hemoglobin A1c, *OGTT* oral glucose tolerance test, *HOMA-IR* homeostatic model assessment-insulin resistance, *eGFR* estimated glomerular filtration rate, *CPK* creatine phosphokinase, *hs-CRP* high-sensitivity C-reactive protein, *HDL* high density lipoprotein, *LDL* low density lipoprotein, *NT-proBNP* N-terminal prohormone of brain natriuretic peptide, *ACE-I* angiotensin converting enzyme inhibitor, *ARB*, angiotensin receptor blocker

### Changes in oxidative stress

**Fig. 1:**
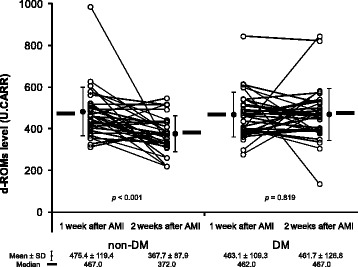
Change in the serum d-ROMs levels after AMI in patients with and without DM. d-ROMs, derivatives of reactive oxygen metabolites; AMI, acute myocardial infarction; DM, diabetes mellitus

Shown in Fig. 1, the d-ROMs level 1 week after AMI did not differ significantly between the DM group and the non-DM group (463.1 ± 109.3 U.CARR vs. 475.4 ± 119.4 U.CARR, respectively, *p* = 0.382). At 2 weeks after AMI, the d-ROMs level had decreased significantly in the non-DM group (from 475.4 ± 119.4 U.CARR to 367.7 ± 87.9 U.CARR, *p* < 0.001) but remained unchanged in the DM group (from 463.1 ± 109.3 U.CARR to 461.7 ± 126.8 U.CARR, *p* = 0.819). Reference d-ROM values are shown in Additional file 2, the value in stable CAD patients without DM was 341.7 ± 101.7 U.CARR, and the value in those with DM was 377 ± 128.3 U.CARR. There was no signifincat difference between these values.

### Major determinant of changes in oxidative stress

**Fig. 2:**
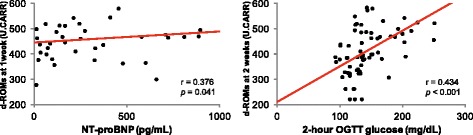
Correlation between d-ROMs levels and NT-proBNP levels at 1 week after AMI (left) and between d-ROMs levels at 2 weeks after AMI and glucose levels at 2 h after OGTT (right). d-ROMs, derivatives of reactive oxygen metabolites; AMI, acute myocardial infarction; NT-proBNP, N-terminal prohormone of brain natriuretic peptide; OGTT, oral glucose tolerance test

In the total patient group, significant positive correlation was found between d-ROMs levels 1 week after AMI and NT-proBNP levels (r = 0.376, *p* = 0.041) (Fig. 2, left panel) and between d-ROMs levels at 2 weeks and 2-h OGTT glucose levels (r = 0.434, *p* < 0.001) (Fig. 2, right panel). There was no relation between the d-ROMs level and age, sex, BMI, glucose profiles except 2-h OGTT glucose level, renal function, CPK, lipids, or use of the various medications. Multivariate logistic regression analysis showed the presence of DM to be a significant predictor of little or no change in the d-ROMs level by 2 weeks after AMI (Table 2), after adjustment for significant factors identified by univariate analysis (odds ratio: 3.33, 95 % confidence interval: 1.15–10.48) (Table 2).

**Table 2:** Factors tested as predictors of lack of change in the d-ROMs level 2 weeks after AMI

	Univariate analysis		Multivariate analysis	
Factor	OR (95 % CI)	*p* value	OR (95 % CI)	*p* value
Age	1.01 (0.95–1.07)	0.677		
Sex	1.50 (0.31–10.87)	0.629		
BMI	1.09 (0.89–1.35)	0.396		
Diabetes mellitus	3.05 (1.07–9.34)	**0.037**	3.33 (1.15–10.48)	**0.027**
Fasting glucose level	1.01 (0.98–1.05)	0.363		
2 h OGTT glucose level	1.02 (1.00–1.03)	**0.032**	1.02 (1.00–1.04)	0.098
HOMA-IR	1.10 (0.79–1.51)	0.570		
eGFR	1.00 (0.98–1.04)	0.675		
CPK (maximum)	1.00 (1.00–1.00)	0.354		
LDL cholesterol	0.99 (0.97–1.01)	0.424		
hs-CRP	1.05 (0.48–2.13)	0.904		
NT-proBNP	1.00 (1.00–1.00)	0.147		
Ca channel blocker	0.18 (0.01–1.11)	0.122		
Beta blocker	3.35 (0.84–17.03)	0.105		
ACE-I/ARB	0.38 (0.02–3.38)	0.428		
Nitrate	0.20 (0.05–0.83)	**0.028**	0.36 (0.07–1.89)	0.216
Statin	0.39 (0.09–1.60)	0.180		

Multivariate logistic regression analysis showed the presence of diabetes mellitus was a significant predictor of no change in the d-ROMs level by 2 weeks after AMI. *d-ROMs* derivatives of reactive oxygen metabolites, *AMI* acute myocardial infarction, OR odds ratio, CI confidence interval, *BMI* body mass index, *OGTT* oral glucose tolerance test, *HOMA-IR* homeostatic model assessment-insulin resistance, *eGFR* estimated glomerular filtration rate, *CPK* creatine phosphokinase, *LDL* low density lipoprotein, *hs-CRP* high-sensitivity C-reactive protein, *NT-proBNP* N-terminal prohormone of brain natriuretic peptide, *ACE-I* angiotensin converting enzyme inhibitor, *ARB* angiotensin receptor blocker

## Discussion

Oxidative stress is implicated in various disorders and pathogeneses. Many studies have shown its involvement in the pathogeneses of lifestyle-related diseases. Previous clinical studies have made use of markers of ROS, such as 8-hydroxydeoxyguanosine and 8-iso-prostaglandin-F2α. However, it is difficult to measure these markers at health checkup facilities. Furthermore, superoxide dismutase, which can serve as an index of antioxidant potential, is also difficult to measure, even at research facilities. In this study, we used a simple assay method to examine the course of oxidative stress between 1 week and 2 weeks after AMI in patients with DM. This is the first study to observe time-specific change in oxidative stress in the early stage after AMI and to examine the difference in exposure to oxidative stress between patients with DM and those without DM.

Recent studies have shown the usefulness d-ROMs assay for evaluating oxidative stress [13–15, 17], and in such evaluation, Trotti et al. found no statistically significant difference between male and female Europeans [11], whereas Fukui et al. found the mean d-ROMs level in female Japanese to be significantly higher than that in male Japanese [25]. Moreover positive correlation between levels of hs-CRP and d-ROMs has been reported [14, 15, 17]. Nevertheless, we found that most clinical characteristics, including sex and hs-CRP, are not factors that significantly influence d-ROMs in patients with AMI. However, we did find that the NT-proBNP level was a significant predictor of the d-ROMs level at 1 week after AMI. This is consistent with the previously reported correlation between BNP and post-MI remodeling [26–28]. Furthermore, DM and hyperglycemia were identified as predictors of non-suppression of ROS production after AMI. Thus, it appears that the d-ROMs level at 1 week is influenced by the effect of MI on the heart itself, whereas the d-ROMs level at 2 weeks is the result of the continuous DM-induced hyperglycemia.

Continuous hyperglycemia increases production of AGEs and high levels of AGEs have been found in the cardiac tissue of diabetic patients [29]. AGEs induce oxidative stress and activate the protein kinase C/diacylglycerol signaling pathway, which is one of the mechanisms by which hyperglycemia exerts adverse cardiovascular effects. In addition, AGEs activate ROS production in mitochondria [20]. Increases in ROS cause cardiac dysfunction by directly damaging proteins and DNA and by inducing apoptosis [30]. We have reported a study in which we treated patients with alpha-glucosidase inhibitor (α-GI) from 1 week to 2 weeks after AMI [31]. In that study, we found that the d-ROMs level in patients treated with α-GI tended to decrease and that endothelial function improved. Thus, oxidative stress plays a pivotal role in the development of the microvascular and cardiovascular complications of DM.

There are some limitations to the present study. First, this was an association study using a case–control design and not randomized, however, the patient characteristics were well matched between groups (Table 1). Second, sample size was small and this was a single-center study. Therefore, we are not able to apply our result to the general population. Third, although we showed reference d-ROM values in stable CAD patients (Additional file 2: Table S1), this was a very short-term follow-up study, and because we did not record d-ROMs levels before AMI or primary PCI and after PCI or more than 2 weeks after AMI, any difference in d-ROMs levels after primary PCI and any progressive change in oxidative stress after AMI in patients with DM remain unknown. Fourth, we excluded patients with heart failure resulting from severe MI, first, because it has been reported that DM can lead to heart failure after MI, and second, because oxidative stress may be high in patients with heart failure [32, 33].

## Conclusions

Our study showed that patients with DM are subject to clinically significant oxidative stress during the first 2 weeks after AMI. Although long-term changes in oxidative stress after AMI in patients with DM remain unknown, results of this short-term follow-up study imply that continuous hyperglycemia drives oxidative stress after AMI, leading to endothelial dysfunction, and progression of atherosclerosis.

## Additional files

Additional file 1: Clinical characteristics of reference patients with stable CAD, per group. (DOCX 21 kb) Additional file 2: d-ROM levels in stable CAD patient with DM and without DM. These data were collected from stable CAD patients with DM and without DM, who had undergone coronary stenting for stable CAD except ACS more than 8 months before data collection. Values are expressed as mean ± SD, and boxes show median and interquartile ranges between the 25th and the 75th percentiles. d-ROM, derivatives of reactive oxygen metabolites; CAD, coronary artery disease; DM, diabetes mellitus; ACS, acute coronary syndrome. (DOCX 385 kb)

## Abbreviations

AGE: advanced glycation end product
AMI: acute myocardial infarction
CAD: coronary artery diesease
CPK: creatine phosphokinase
DM: diabetes mellitus
d-ROM: derivatives of reactive oxygen metabolite
eGFR: estimated glomerular filtration rate
HbA1c: hemoglobin A1c
HOMA-IR: homeostasis model assessment of insulin resistance
hs-CRP: high-sensitivity C-reactive protein
NT-proBNP: N-terminal prohormone of brain natriuretic peptide
OGTT: oral glucose tolerance test
PCI: percutaneous coronary intervenetion
ROS: reactive oxygen species.
α-GI: alpha-glucosidase inhibitor
