# Supplementary material for: A comparative study of time-specific oxidative stress after acute myocardial infarction in patients with and without diabetes mellitus
Source: BMC Cardiovasc Disord. 2016 May 23;16:102. doi: 10.1186/s12872-016-0259-6 (PMC4877735; doi:10.1186/s12872-016-0259-6)
Supplement: Additional file 1: — Clinical characteristics of reference patients with stable CAD, per group. (DOCX 21 kb) [file 12872_2016_259_MOESM1_ESM.docx]

**Supplemental Table Clinical characteristics of reference patients with CAD, per group.**

|  | DM | non DM | p value |
| --- | --- | --- | --- |
|  | n = 28 | n = 40 |  |
| Age (years) | 65.7 ± 10.9 | 65.2 ± 9.8 | 0.900 |
| Gender, male (%) | 22 （78.6） | 34 (85.0) | 0.618 |
| BMI | 24.4 ± 3.6 | 22.0 ± 2.4 | 0.062 |
| Period after PCI | 8.5 ± 3.0 | 9.9 ± 2.9 | 0.166 |
| **Risk factor** |  |  |  |
| Hypertention, n (%) | 24 (85.7) | 26 (65.0) | 0.151 |
| Dyslipidemia, n (%) | 25 (89.3) | 23 (57.5) | **0.026** |
| Smoking, n (%) | 21 (75.0) | 19 (47.5) | 0.070 |
| **Biochemical markers** |  |  |  |
| HbA1c (%) | 7.3 ± 1.4 | 5.6 ± 0.3 | **<0.001** |
| eGFR (ml/min./1.73m^2^) | 65.9 ± 23.1 | 68.2 ± 17.2 | 0.501 |
| hs-CRP (mg/dl) | 0.062 ± 0.022 | 0.117 ± 0.100 | 0.193 |
| LDL-Cholesterol (mg/dl) | 83.9 ± 23.7 | 91.9 ± 31.3 | 0.469 |
| **Medications** |  |  |  |
| Ca channel blocker, n (%) | 13 (46.4) | 22 (55.0) | 0.605 |
| Beta blocker, n (%) | 17 (60.7) | 12 (30.0) | 0.060 |
| ACE-I/ARB, n (%) | 19 (67.9) | 24 (60.0) | 0.641 |
| Nitrates, n (%) | 20 (61.4) | 33 (82.5) | 0.354 |
| Statin, n (%) | 21 (75.0) | 29 (72.5) | 0.768 |
| Sulfonylurea, n (%) | 9 (32.1) | - |  |
| Biguanide, n (%) | 7 (25.0) | - |  |
| Thiazolidine, n (%) | 4 (14.3) | - |  |
| α-GI, n (%) | 5 (17.9) | - |  |
| Glinide, n (%) | 1 (3.6) | - |  |

Data are expressed as mean ± SD or number (%). P value was obtained by ANOVA or chi-square test. DM, diabetes mellitus; BMI, body mass index; PCI, percutaneous coronary intervention; HbA1c, hemoglobin A1c; eGFR, estimated glomerular filtration rate; hs-CRP, high-sensitivity C-reactive protein; LDL, low density lipoprotein; ACE-I, angiotensin converting enzyme inhibitor; ARB, angiotensin receptor blocker; α-GI, alpha glucosidase inhibitor.
