# Supplementary figures and images for: A comparative study of time-specific oxidative stress after acute myocardial infarction in patients with and without diabetes mellitus
Source: BMC Cardiovasc Disord. 2016 May 23;16:102. doi: 10.1186/s12872-016-0259-6 (PMC4877735; doi:10.1186/s12872-016-0259-6)

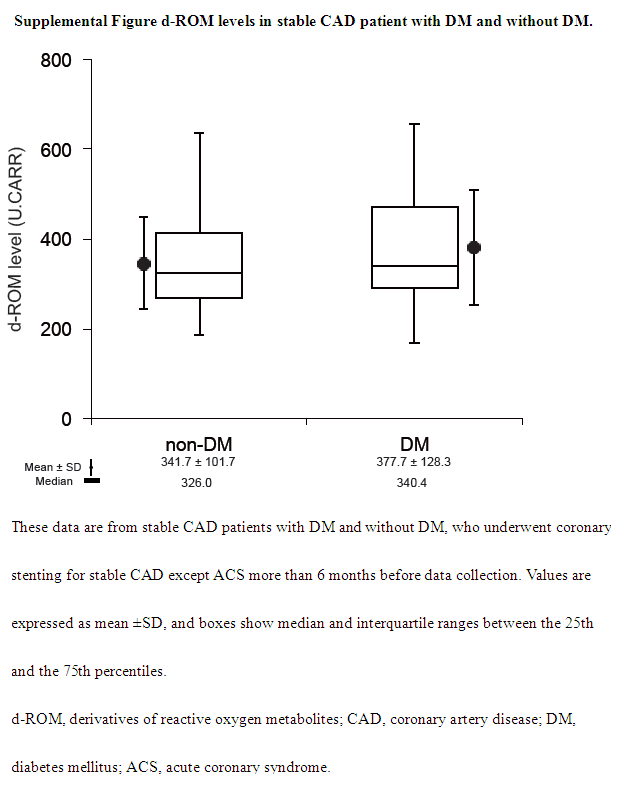

Supplement: Additional file 2: — d-ROM levels in stable CAD patient with DM and without DM. These data were collected from stable CAD patients with DM and without DM, who had undergone coronary stenting for stable CAD except ACS more than 8 months before data collection. Values are expressed as mean ± SD, and boxes show median and interquartile ranges between the 25th and the 75th percentiles. d-ROM, derivatives of reactive oxygen metabolites; CAD, coronary artery disease; DM, diabetes mellitus; ACS, acute coronary syndrome. (DOCX 385 kb) [file 12872_2016_259_MOESM2_ESM.docx]
